# Differential Expression Patterns of TDP-43 in Single Moderate versus Repetitive Mild Traumatic Brain Injury in Mice

**DOI:** 10.3390/ijms222212211

**Published:** 2021-11-11

**Authors:** Tamara Janković, Petra Dolenec, Jelena Rajič Bumber, Nika Gržeta, Jasna Kriz, Gordana Župan, Kristina Pilipović

**Affiliations:** 1 Department of Basic and Clinical Pharmacology and Toxicology, Faculty of Medicine, University of Rijeka, 51 000 Rijeka, Croatia; tamara.jankovic@medri.uniri.hr (T.J.); petra.dolenec@medri.uniri.hr (P.D.); jelena.rajic@medri.uniri.hr (J.R.B.); nika.grzeta@medri.uniri.hr (N.G.); gordana.zupan@medri.uniri.hr (G.Ž.); 2 Department of Psychiatry and Neuroscience, Faculty of Medicine, Université Laval, Quebec City, QC G1V 0A6, Canada; jasna.kriz@fmed.ulaval.ca

**Keywords:** mice, microglia, NF-κ B, postsynaptic density protein 95, traumatic brain injury, transactive response DNA-binding protein 43

## Abstract

Traumatic brain injury (TBI) is a disabling disorder and a major cause of death and disability in the world. Both single and repetitive traumas affect the brain acutely but can also lead to chronic neurodegenerative changes. Clinical studies have shown some dissimilarities in transactive response DNA binding protein 43 (TDP-43) expression patterns following single versus repetitive TBI. We explored the acute cortical post-traumatic changes of TDP-43 using the lateral fluid percussion injury (LFPI) model of single moderate TBI in adult male mice and investigated the association of TDP-43 with post-traumatic neuroinflammation and synaptic plasticity. In the ipsilateral cortices of animals following LFPI, we found changes in the cytoplasmic and nuclear levels of TDP-43 and the decreased expression of postsynaptic protein 95 within the first 3 d post-injury. Subacute pathological changes of TDP-43 in the hippocampi of animals following LFPI and in mice exposed to repetitive mild TBI (rmTBI) were studied. Changes in the hippocampal TDP-43 expression patterns at 14 d following different brain trauma procedures showed pathological alterations only after single moderate, but not following rmTBI. Hippocampal LFPI-induced TDP-43 pathology was not accompanied by the microglial reaction, contrary to the findings after rmTBI, suggesting that different types of brain trauma may cause diverse pathophysiological changes in the brain, specifically related to the TDP-43 protein as well as to the microglial reaction. Taken together, our findings may contribute to a better understanding of the pathophysiological events following brain trauma.

## 1. Introduction

Traumatic brain injury (TBI) is one of the most common causes of disability and death in younger individuals [1,2,3]. It was once predicted to surpass many other conditions as a major cause of death and disability in an overall population by the year 2020 [4]. TBI occurs as a result of the action of the mechanical force to a person’s head, causing cerebral damage which results in permanent neurological and behavioral consequences and therefore requires continuous health care [5]. The majority of patients hospitalized due to brain trauma are diagnosed with mild TBI, while moderate and severe TBIs are rarer, with an overall ratio of 22:1.5:1 [2]. Moderate and severe TBIs are typically consequences of traffic accidents or falls [3]. Very commonly, mild injuries occur multiple times, particularly in athletes involved in contact sports [6,7], the military personnel [8,9], and the victims of domestic violence [10].

Both single and repetitive exposures to traumatic injury may affect the brain acutely and can lead to chronic neurodegenerative changes, such as chronic traumatic encephalopathy (CTE), a degenerative brain disorder found in individuals with a history of repetitive brain traumas and associated with behavioral problems and the development of dementia [11]. Even though CTE is commonly believed to be the result of exposure to multiple head traumas during a person’s life, certain studies point to a possible connection of single moderate TBI and the development of chronic neuropathological findings [12,13]. In most cases of CTE, pathological modifications of transactive response (TAR) DNA-binding protein 43 (TDP-43) are found. TDP-43 is a ubiquitous DNA and RNA binding protein located dominantly in the nucleus with functions in alternative splicing, microRNA biogenesis, promoting mRNA stability, translational regulation, and nuclear pore transport [14,15]. It is well known that, in the amyotrophic lateral sclerosis (ALS) and frontotemporal lobar degeneration (FTLD), TDP-43 translocates from the nucleus of neurons and glia and forms phosphorylated, ubiquitinated and proteolytically-cleaved deposits in the cytoplasm [7,15,16,17,18]. Overexpression of human or mouse TDP-43 in in vivo assays have showed that overexpressed TDP-43 accumulates in the cytoplasm, with animals developing phenotypes similar to FTLD [15]. It has also been detected that TDP-43 is upregulated in the spinal cord after axotomy [19] and in the cortex and hippocampus following repetitive mild TBI (rmTBI) in the mouse [20,21].

Previous studies have also shown that inflammation promotes mislocalization and aggregation of TDP-43 in vitro and in vivo [22]. Swarup et al. [23] first proposed that TDP-43 accumulation, at least in part, stimulates nuclear factor kappa beta (NF-κB) activation. Besides the proinflammatory effect, NF-κB also has a protective role, and it is included in the processes of synaptic plasticity and memory [24,25,26,27]. The upregulation of NF-κB has been shown in various TBI models [28,29,30,31,32], but its interdependence with TDP-43 in the experimental brain trauma has not been explored thus far. One of the NF-κB target genes is the postsynaptic density protein 95 (PSD-95) [33,34], a scaffold protein responsible for the regulation of dendritic spines, presynaptic and postsynaptic proteins, and glutamate receptor trafficking [35]. Regarding the role of TDP-43 on synaptic functions, opposite explanations have been offered. Specifically, in the cortex of transgenic TDP-43^A315T^ mice, the synaptic function is impaired due to the development of mushroom spines with lower efficiency [36]; however, in the transgenic TDP-43^Q331K^ animals, increased synaptic activity in the mouse motor cortex was observed [37]. Furthermore, it has also been found that, in rodent brains and hippocampal neuronal cultures, TDP-43 is present in RNA granules in the dendrites where it possibly functions as a synaptic activity-responsive factor [38,39,40]. Consequently, it has been suggested that the impairment of the TDP-43-mediated neuronal plasticity could be connected to the development of some neurodegenerative diseases, including FTLD [40,41,42].

In clinical studies, TDP-43 proteinopathy was evident after a single TBI but it was revealed that it has different characteristics in comparison to the postmortem findings from the individuals that have suffered multiple TBIs during life [43]. The aim of our study was to gain insight into the single moderate trauma-induced TDP-43 changes in the mouse cerebral cortex in the early post-injury time points, as well as to investigate if there is a correlation between the post-traumatic TDP-43 dysregulation and the changes in the expression of NF-κB and PSD-95. We also explored whether there is a difference in the TDP-43 pathology in the hippocampus, a brain region somewhat remote from the cortex, directly affected brain structure, between the mice subjected to single moderate versus repetitive mild brain trauma at 14 d after the trauma procedures. In the animals of these experimental groups, in the same brain structure and at the matching subacute time point, we also investigated if there are changes in some markers of inflammation and microglial activation.

Here, we report the marked pathophysiological differences in the brain’s response to injury following single and/or repetitive trauma. A single moderate TBI causes early translocation of TDP-43 from the nucleus to the cytoplasm in the mouse ipsilateral cerebral cortex. Cytoplasmic TDP-43 mislocalization was found in neurons and microglial cells, but not astrocytes. An acute decrease in the cortical PSD-95 levels was also observed, as well as a correlation between the cellular presence of this synaptic protein and the increased cytoplasmic TDP-43 expression. Furthermore, at 14 d post-injury, we detected some pathological changes of TDP-43 in the hippocampi of animals subjected to single moderate, but not repetitive, mild brain trauma. Changes in the hippocampal TDP-43 expression in mice following single moderate head trauma were not accompanied by a significant microglial reaction, contrary to findings in the repetitively injured animals. These pathophysiological differences in single and repetitive brain trauma could be of further importance.

## 2. Results

### 2.1. Single Moderate Traumatic Brain Injury Causes Changes in the TDP-43 Expression Pattern in the Cortical Neurons and Microglia of the Injured Mice

**Figure 1:**
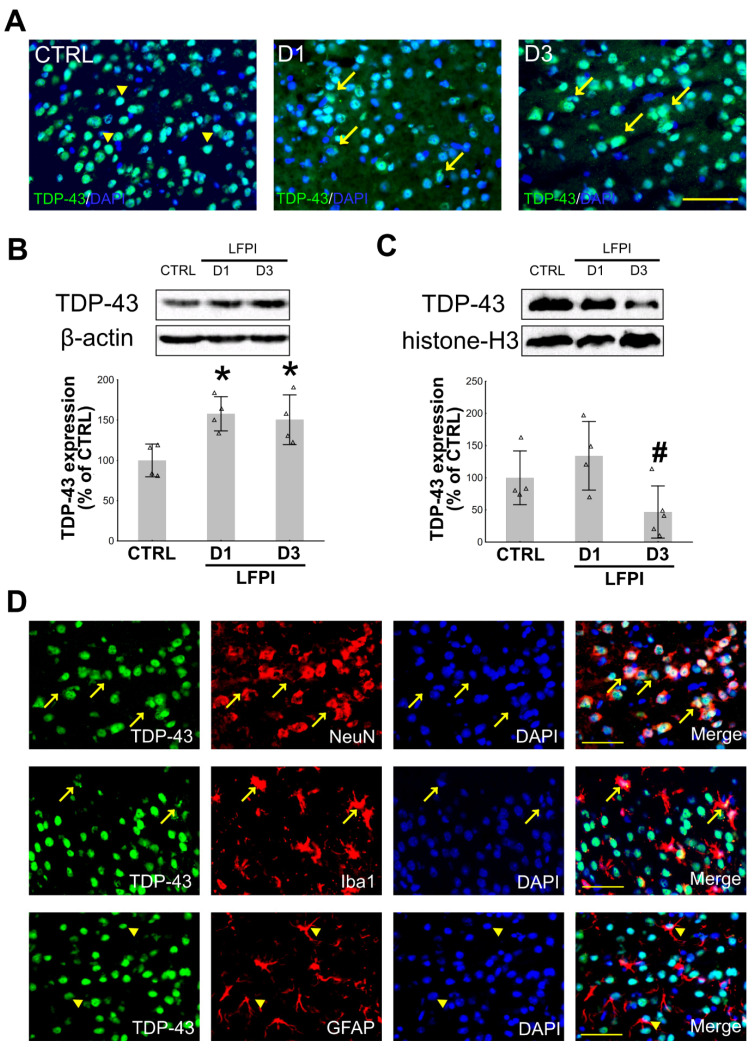
Subcellular localization of transactive response (TAR) DNA binding protein 43 (TDP-43) in the mouse ipsilateral cortices 1 and 3 d after a single lateral fluid percussion injury (LFPI): (**A**) Representative microphotographs show TDP-43-immunostained (green) sections, counterstained with DAPI nuclear stain (blue) in the animal of the control group (CTRL), sacrificed 1 day after the experimental procedures, and the mice with LFPI sacrificed 1 (D1) or 3 (D3) d following single moderate LFPI. Arrowheads point to cells with predominant nuclear staining, and arrows point to cells with pronounced cytoplasmic TDP-43 expression. Scale bar: 100 µm. (**B**,**C**) Representative immunoblots of TDP-43, β-actin, and histone-H3 and the bar plots of their corresponding densitometry analyses of the cytoplasmic (**B**) and the nuclear (**C**) TDP-43 expression levels in the animals of the control group (CTRL) and the injured mice sacrificed at different time points following LFPI. In the densitometric analyses, TDP-43 results were corrected for the values of β-actin (cytoplasmic loading control) or histone-H3 (nuclear loading control) and were expressed as a % of the related control groups. Error bars represent ± *SD* (*n* = 4–5 mice per group). Each triangle represents data from an individual mouse. * *p* < 0.05; significantly different from the related control group. # *p* < 0.05; significantly different from the LFPI D1 group. (**D**) Representative microphotographs of the ipsilateral cortices, double labeled with anti-TDP-43 antibody (green) and the markers of neurons (NeuN), microglia (Iba1) or astrocytes (GFAP) (all red), from the animal sacrificed 3 d following single moderate LFPI. Sections were counterstained with DAPI nuclear stain (blue). Arrows point to cells with prominent cytoplasmic, and arrowheads indicate cells with dominant nuclear TDP-43 expression. Scale bar: 100 μm.

Growing data suggest an important role of TDP-43 in the acute stage of neurodegeneration following different types of brain injury, including brain trauma [19,43,44,45,46,47]. TDP-43 cellular mislocalization has been previously described in several studies, in which different models of TBI, varying injury severities, and experimental animal species were used [21,44,45,46,48,49,50]. Here, we aimed to assess the dynamics of the changes in the cytoplasmic and nuclear TDP-43 protein expressions in the mouse cortices at different post-trauma time points following single moderate LFPI as it is shown in Figure 1 and Figure S1.

In the ipsilateral cortices of injured animals, increased cytoplasmic TDP-43 immunostaining was evident at both day 1 and day 3 after the brain trauma compared to the control mouse, in which this protein was mostly situated in the cell nuclei (Figure 1A). Densitometric analyses of the TDP-43 Western blots (Figure 1B,C) revealed a significant increase in its cytoplasmic content compared to the results from the cortical samples of the control animals at both 1 and 3 post-injury d [F(2,9) = 6.525; *p* = 0.018]. A decrease in the nuclear expression of this protein was detected at day 3 following LFPI but only compared to the day 1 level [F(2,10) = 4.302; *p* = 0.045]. Further, we wanted to determine whether these TDP-43 expression changes are cell type specific, i.e., if the increased cytoplasmic presence of this protein could be detected in the ipsilateral cortical neurons, microglia, or astrocytes after LFPI. Immunofluorescence analysis of the ipsilateral cortices of injured mice (Figure 1D) showed increased cytoplasmic TDP-43 staining in neurons and microglia; however, in our experiments, in the control and the injured animals, cytoplasmic expression of TDP-43 in the astrocytes was not detected.

At this point, it was unclear whether the observed changes in the TDP-43 expression that we found in the ipsilateral cortices of injured animals was part of the physiological response to injury or if it was a sign of pathological changes. Therefore, we wanted to determine whether LFPI is also associated with the formation of phosphorylated TDP-43 and its fragments in the mouse ipsilateral cortex at 1 and 3 d following trauma. As shown in the Figure S2, we did not detect any additional TDP-43 abnormalities in the context of single LFPI. 

Taken together, these results reveal the changes in the TDP-43 subcellular expression at the investigated time points following a single moderate TBI. This cytoplasmic TDP-43 mislocalization was not only restricted to neurons but was also found in microglial cells. 

### 2.2. Single Moderate Brain Trauma Triggers Changes in the Cortical Expression of p65 Subunit of NF-κB That Are Area- and Cell Type-Dependent

In the previous studies, it was shown that TDP-43 may act as a co-activator of the p65 subunit of NF-κB in the models of ALS [23] and stroke [47], and that it is involved in the proinflammatory genes’ transcription as well as in the inflammation-mediated neurodegeneration [23]. Here, we first aimed to explore the NF-κB expression changes in the ipsilateral cortices of injured mice acutely following a single moderate LFPI.

**Figure 2:**
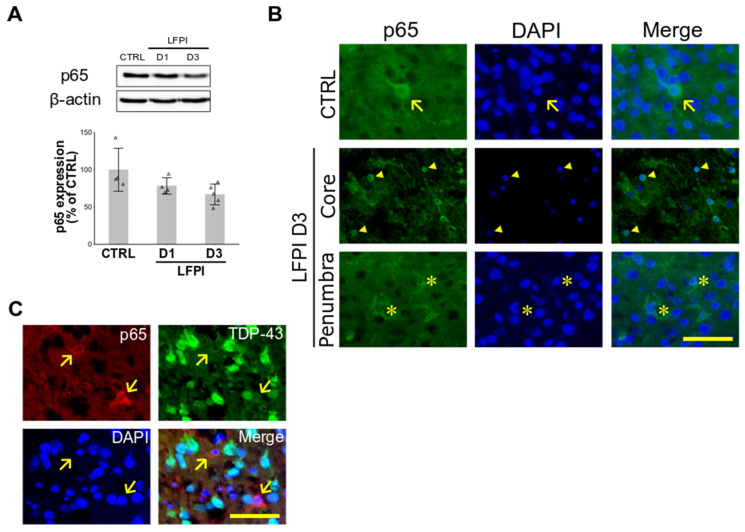
Changes in the cortical cytoplasmic expression of p65 subunit of nuclear factor kappa B (NF-κB) following single moderate lateral fluid percussion injury (LFPI) in the mice: (**A**) Representative p65 and β-actin immunoblots and the bar plots of their corresponding densitometry analyses in the animals of the control group (CTRL) and the mice with brain trauma sacrificed at 1 (D1) or 3 (D3) d following LFPI are shown. In the densitometric analyses, p65 results were corrected for the values of β-actin and expressed as % of the related control group. Error bars represent ± *SD* (*n* = 4–5 mice per group). Each triangle represents data from an individual mouse. (**B**) Immunofluorescence of the ipsilateral cortex of the control mouse and the injured animal sacrificed at 3 d after trauma using the p65 antibody. In the injured mouse, microphotographs from the core traumatized area and the pericontusional penumbra are presented. Arrow points to a cell with neuron-like morphology and pronounced p65 cytoplasmic staining. Arrowheads point to cells characterized with nuclear translocation of p65 subunit of NF-κB. Asterisks denote glia-like, p65 positive cells. (**C**) Double immunofluorescence of the ipsilateral cortex section from the brain-injured mouse sacrificed at 3 d following LFPI using the p65 (red) and TDP-43 (green) antibodies. Arrows point to cells with diminished nuclear TDP-43 signal and pronounced p65 cytoplasmic staining. All the immunofluorescent sections were counterstained with DAPI nuclear stain (blue). Scale bars: 50 μm.

Representative immunoblots and the densitometric analyses of the expression levels of the cytoplasmic p65 subunit of NF-κB are shown in Figure 2A. Statistical analysis was conducted to examine the variances in the p65 expression levels between the experimental groups and no significant differences were found, even though a trend of a decrease in the p65 expression in mice with LFPI was apparent, especially at day 3 following LFPI [F(2,10) = 3.451; *p* = 0.072]. Next, using the immunofluorescence method, we detected variances in the p65 staining pattern, depending on the part of the examined cortex of injured mice (Figure 2B). Specifically, in the core area of the injured cortex, p65 staining was dominantly evident in the cell nuclei suggesting translocation of this protein and in the activation of the NF-κB pathway. However, in the pericontusional, i.e., the penumbral area, we detected increased cytoplasmic expression of this protein in cells with glia-like morphology.

Since it has been previously suggested that deregulation of TDP-43 might potentiate NF-κB-mediated pathogenic pathways [23,47], we wanted to see whether there is an association between the p65 and the TDP-43 staining patterns in the ipsilateral cortices of animals acutely following LFPI. Double immunofluorescence of the brain cortex of traumatized animals sacrificed at 3 d following LFPI was conducted, and a representative section is presented in Figure 2C. In the ipsilateral cortices of injured animals, we detected cells with pronounced p65 cytoplasmic staining and specific glia-like morphologic features in which there was missing or very weak nuclear TDP-43 staining. 

In summary, these findings suggest area and cell type-dependent changes in the expression of the p65 subunit of NF-κB in the traumatized mouse cortex and, additionally, the cell-type dependent correlation between the subcellular p65 and TDP-43 expression patterns.

### 2.3. Single Moderate Brain Trauma Causes a Decrease in Postsynaptic Protein PSD-95 Levels in the Ipsilateral Cortices of Injured Animals

**Figure 3:**
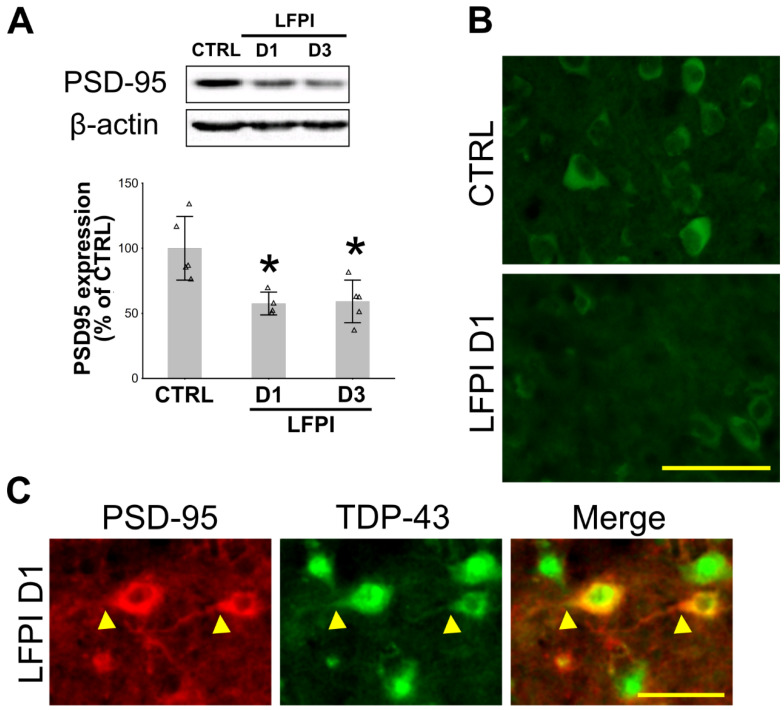
Changes in the cortical expression of the postsynaptic density protein 95 (PSD-95) at 1 and 3 d following single moderate lateral fluid percussion injury (LFPI) in mice: (**A**) Representative immunoblots of PSD-95 and β-actin and the bar plots of their corresponding densitometric analyses in animals sacrificed at d 1 (D1) or 3 (D3) after trauma and in the mice of the control group (CTRL) are shown. In the densitometric analyses, PSD-95 results were corrected for the values of β-actin and presented as % of the related control group. Error bars represent ± *SD* (*n* = 4–5 mice per group). Each triangle represents data from an individual mouse * *p* < 0.05; significantly different from the control group. (**B**) Representative microphotographs show PSD-95-immunostained ipsilateral cortical sections in the animal of the control group and the mouse sacrificed 1 day after the LFPI. Scale bar: 50 μm. (**C**) Double immunofluorescence of the ipsilateral cortex section from the injured mouse sacrificed 1 day following LFPI using the PSD-95 (red) and TDP-43 (green) antibodies. Arrowheads point to cells with increased cytoplasmic TDP-43 staining and pronounced PSD-95 expression. Scale bar: 50 μm.

Since it has been established that TDP-43 has an important role in processing RNA for proteins involved in synaptic transmission and plasticity [39,40], we next hypothesized that, in mice with a single moderate TBI, there is a correlation between the cytoplasmic levels of this protein and the levels of the postsynaptic protein PSD-95. Therefore, we first examined whether PSD-95 expression in the cortices of injured mice is affected by the LFPI. Western blotting was performed on the cortical samples from the mice of the control group and the animals sacrificed at 1 or 3 d following LFPI. The statistical analyses revealed a significant decrease in PSD-95 expression levels in the ipsilateral cortices of traumatized mice at both post-injury time points in comparison to the results from the control group [F(2,11) = 8.274, *p* = 0.006] (Figure 3A). PSD-95 immunofluorescence was performed, and we detected a decrease in the immunofluorescent signal in the mouse ipsilateral cortical tissue after LFPI (Figure 3B). The observed changes in the PSD-95 expression levels coincided with a significant loss of neurons, as shown in Figure S3, and with a decrease in the NeuN staining, i.e., post-traumatic loss of cortical neurons (Figure S4).

Double immunofluorescence using the TDP-43 and the PSD-95 antibodies (Figure 3C) revealed only a few scattered PSD-95 positive cells in the cortical injury area, but those cells also show the increased presence of cytoplasmic TDP-43. Notably, TDP-43 was not only present in the cell body, but also in the axons in which it co-localized with PSD-95. 

### 2.4. Single Moderate, but Not Repetitive Mild Traumatic Brain Injury Induces Pathological Post-Translational Changes of TDP-43 in the Mouse Hippocampi

Since the results of the clinical studies suggest a difference in the TDP-43 expression changes following single or repetitive TBI [43,51], particularly related to the chronic posttraumatic sequelae, we next wanted to test whether this was the case in the experimental models of these two types of brain trauma. In that regard, we used the LFPI model for the single moderate TBI, and for the repetitive mild brain trauma, we exposed mice to mild TBIs twice daily for 5 consecutive d. Injured mice were sacrificed 14 d following LFPI, i.e., 14 d after the last mild brain trauma in the rmTBI experiment (details regarding the experimental procedures are described in the Materials and Methods section, and presented in Figure 6). We specifically aimed to explore whether there were signs of pathological changes in the hippocampus, a cerebral region somewhat remote from the cortex, but significantly exposed to damage by TBI [52]. By focusing on this neuroanatomical region and the 14 d’ time point, we also aimed to determine whether there is a progression of the TDP-43 proteinopathy from the ipsilateral cortex to other brain areas in the subacute time period after different TBI procedures. 

**Figure 4:**
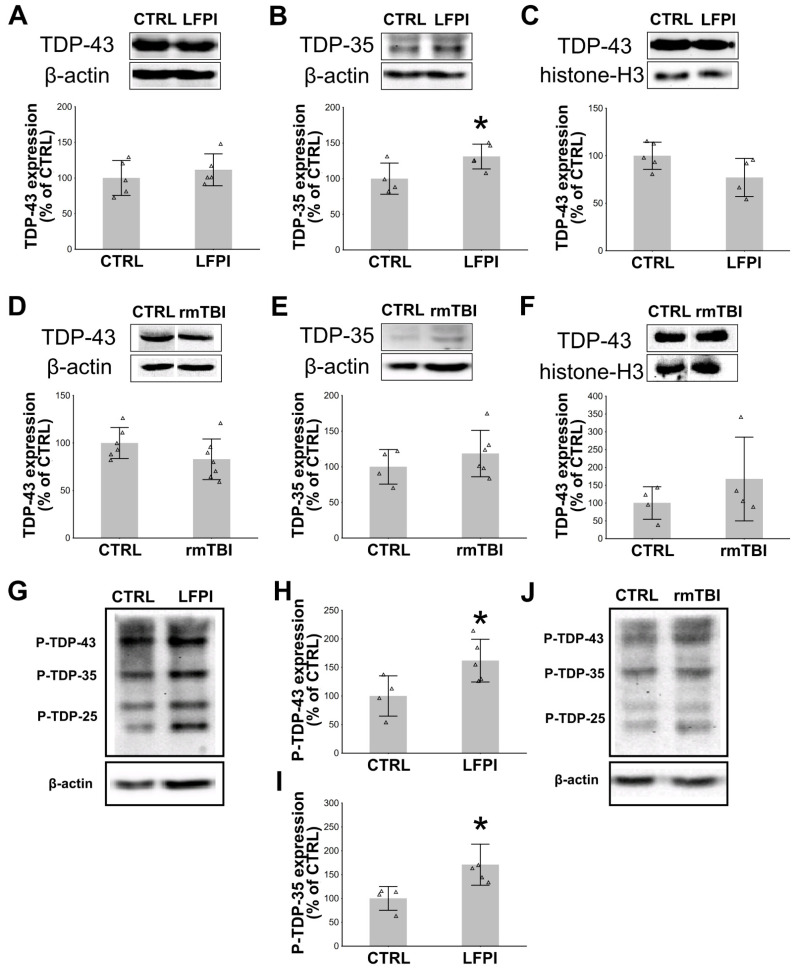
Effects of single moderate lateral fluid percussion injury (LFPI) or repetitive mild traumatic brain injury (rmTBI) on the subcellular localization and cleavage of transactive response (TAR) DNA binding protein 43 (TDP-43) and the production of phosphorylated TDP-43 species in the mouse hippocampi at 14 d after the experimental brain trauma protocols: (**A**–**F**) Representative immunoblots of TDP-43, β-actin, and histone-H3 and the bar plots of their corresponding densitometric analyses of the cytoplasmic (**A**,**D**) and the nuclear (**C**,**F**) TDP-43 expression levels in the animals of the related control groups (CTRL) and mice sacrificed at 14 d after LFPI or the last mild trauma. Additionally, analysis of the cytoplasmic TDP-35 expression was done in the hippocampal samples from the animals exposed to LFPI (**B**) or rmTBI trauma procedures (**E**). (**G**–**J**) Western blotting analysis of the hippocampal cytosolic lysates using the phospho-TDP-43 antibody in animals sacrificed 14 d following LFPI or the rmTBI procedures. Representative immunoblots of the phosphorylated TDP-43 (P-TDP-43), TDP-35 (P-TDP-35) and β-actin are shown for the mice from both LFPI (**G**) and the rmTBI (**J**) experiments. Bar plots showing densitometric analysis of the hippocampal cytoplasmic expression of the phosphorylated TDP-43 (P-TDP-43) (**H**) and TDP-35 (P-TDP-35) (**I**) is presented only for the animals sacrificed at 14 d after LFPI experiment. Neither LFPI nor rmTBI caused a significant change in the levels of the phosphorylated 25 kDa fragment of TDP-43 (P-TDP-25). In all the densitometric analyses, the results were corrected for the values of β-actin (cytoplasmic loading control) or histone-H3 (nuclear loading control) and expressed as % of the related control groups. Error bars represent ± *SD* (*n* = 4–7 mice per group). Each triangle represents data from an individual mouse. * *p* < 0.05; significantly different from the related control group.

In the animals with LFPI, both cytoplasmic (Figure 4A) and nuclear TDP-43 (Figure 4C) expressions in the hippocampi did not differ from the levels detected in the control animals. However, as further revealed in Figure 4B, LFPI was associated with a significant increase in the hippocampal expression of the cleaved pathogenic cytoplasmic TDP-35 fragments when compared to the control animals (*p* = 0.049). Next, we investigated the TDP-43 expression patterns following rmTBI. The Western blot analyses showed no difference in cytoplasmic (Figure 4D) and nuclear TDP-43 (Figure 4F) levels when compared to related controls. Unlike the increase observed following LFPI (Figure 4B), we did not observe pathological fragmentation of TDP-43 14 d after the last mild TBI (Figure 4E).

Next, we wanted to test whether the LFPI or rmTBI may trigger other post-translational changes of the investigated protein, specifically phosphorylation of TDP-43 and its fragments. As revealed in Figure 4G–J, in the hippocampi of mice with LFPI, densitometric analyses of blots immunolabeled with specific phospho(409/410)-TDP-43 antibody revealed a significant increase in the phosphorylated TDP-43 levels (Figure 4H) (*p* = 0.039). Levels of the phosphorylated TDP-35 (Figure 4I) (*p* = 0.023) in the ipsilateral hippocampus after single moderate LFPI were also higher in comparison to the related control group, as seen in representative immunoblots (Figure 4G). In our experimental conditions, repetitive brain traumas did not affect the formation of the phosphorylated TDP-43 and its cleaved species in the investigated brain region (blot shown in Figure 4J is representative for *n* = 4 animals/group).

These results suggest that, in the hippocampi of the traumatized animals, at this subacute post-injury time point, there were no significant changes in the overall cytoplasmic or nuclear content of the full-length TDP-43 protein following these two distinct types of TBI. Contrary, a significant difference between these brain trauma models is detectable due to the presence of the pathogenic 35 kDa fragments in the mouse hippocampi following only single moderate TBI. Additionally, in the same brain region, LFPI, but not rmTBI, triggered phosphorylation of TDP-43 and its 35 kDa cleavage product. We did not detect significant changes in the expression of the 25 kDa cleavage product of TDP-43 or its phosphorylated form when comparing LFPI- or rmTBI-exposed animals to their respective control groups (data not shown). 

### 2.5. Post-Translational Modifications of TDP-43 Protein in the Hippocampus of Mice Exposed to a Single Moderate Traumatic Brain Injury Are Not Accompanied by Changes in the Microglia Activation Pattern

After detecting the differences in the presence of the pathological TDP-35 fragment as well as in the appearance of P-TDP-43 and P-TDP-35 in the mouse hippocampi after LFPI in comparison to rmTBI animals, at 14 d following the experimental procedures, we wanted to determine whether these changes are related to microglial dysregulation in the same brain region. Namely, it has been suggested that the immune activation, including the activation of the microglia, the brain’s immune system cells, could result from an initial pathological insult. This triggering event could consequently cause an ongoing cytotoxic response, including the accumulation of TDP-43 [42], and result in secondary chronic neuroinflammation. 

In order to test our hypothesis, we evaluated the hippocampal levels of some protein markers of microglia activity by using Western blotting. Densitometric analyses of the protein blots revealed that, both in the LFPI and the rmTBI experiments, there were no changes in the hippocampal levels of the investigated microglial markers, specifically Iba1, iNOS, TLR-2, CD86, and CD206, at 14 d after both brain trauma protocols in relation to their designated control groups (Figure S5). 

Considering that this molecular approach to phenotypic characterization of microglia activation has garnered much criticism in recent years as being too simplistic and reductive [53], we also examined whether the investigated brain trauma types potentially cause morphological changes of the hippocampal microglia.

**Figure 5:**
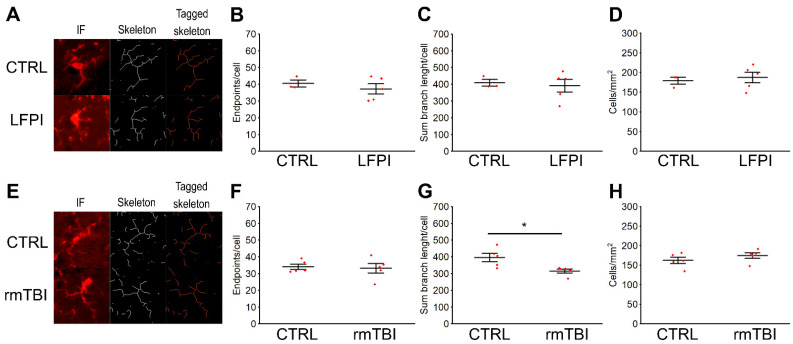
Effects of single moderate lateral fluid percussion injury (LFPI) or repetitive mild traumatic brain injury (rmTBI) on the microglial activation pattern in the hippocampal dentate gyrus: Analysis of microglial morphology and the number of cells was done on hippocampal sections that were stained by immunofluorescence (IF) with the anti-Iba1 antibody. Images are representative of 3–5 mice per group. The cells were converted to their skeleton form (**A**,**E**) followed by the analysis of the number of branches (endpoints) per cell (**B**,**F**) and sum branch (process) length per cell (**C**,**G**). Results show that the microglia in the dentate gyrus of the hippocampus of mice sacrificed at 14 d following the last mild trauma in the rmTBI experiment have significantly reduced sum branch length compared with control mice, implying a more amoeboid reactive morphology. In both LFPI and rmTBI experiments, the total number of microglial cells per mm2 in the investigated brain region did not differ from the data from the respective control groups (**D**,**H**). Each dot represents data from an individual mouse. Data are pooled from at least six sections from 3–5 mice per group. * *p* < 0.05; significantly different from the related control group.

In Figure 5, the results of the quantitative analyses of the number and morphology of microglial cells in the hippocampal dentate gyri are shown. In animals injured by a single moderate trauma and in the repetitively injured mice, the microglial cell count in the investigated region did not significantly differ from the measurements determined in the related control mice (LFPI: *p* = 0.679; rmTBI: *p* = 0.299) (Figure 5D,H). However, in the rmTBI animals, we detected some modest morphological changes of microglia. Specifically, rmTBI caused a decrease in the average sum branch length per cell (*p* = 0.020) (Figure 5G), which was not the case with the findings in the LFPI mice (*p* = 0.744) (Figure 5C). In the investigated brain region of mice exposed to either LFPI or rmTBI, the number of endpoint processes per cell remained unchanged (LFPI: *p* = 0.481; rmTBI: *p* = 0.792) (Figure 5B,F). In conclusion, this finding suggests that the exposure to repetitive head impacts induces de-ramification of microglia in the hippocampus, i.e., transformation from ramified into amoeboid morphology, which is considered to be one of the earliest manifestations of microglial activation.

Given the fact that no change in the hippocampal expression of the pathological TDP-43 species was observed in the rmTBI group of animals, we argue that, at this subacute time point, changes in the microglia activation state are unrelated to TDP-43 protein function and changes. This is also supported by the absence of correlation between the presence of the pathological TDP-35 fragment, the appearance of P-TDP-43 and P-TDP-35, and the microglia state in the hippocampi of mice subjected to LFPI.

## 3. Discussion

In this study, we provide evidence from in vivo experiments on some changes in the cortical TDP-43 expression patterns as well as in the levels of the p65 subunit of NF-κB and in the postsynaptic protein PSD-95 in a mouse model of single TBI of moderate severity. We used a model that resulted in brain trauma with characteristics similar to the injuries suffered in traffic accidents or falls. We also detected, in the subacute post-injury period, some differences in the hippocampal expression of the pathogenic TDP-35 fragment, levels of phosphorylated full-length TDP-43 and its 35 kDa cleaved fragment, as well as in the microglial reactivity between the aforementioned model and a TBI model representative of the type of repetitive brain trauma, typical for contact sports or in the military personnel. 

The brain trauma models we chose for this study represent one of the most used TBI models for their respective types of brain injury and, in both of them, neurobehavioral and cognitive deficits, regularly observed in patients with brain trauma, are detected [54,55]. However, there may be some differences in the clinical presentation of the neurodegenerative changes found in patients who experienced different types of TBI during life. Namely, cognitive and neurobehavioral sequelae in patients following repetitive TBI are viewed as part of the CTE diagnosis [12]. Regarding the chronic consequence of a single TBI, it is considered to be the underlying cause of specific neurodegenerative diseases such as Alzheimer’s disease (AD) [56]. Nevertheless, research suggests a certain overlap of clinical presentations of neurological disorders regardless of the type of TBI the patients suffered earlier in life [12,57,58].

In the animals subjected to a single moderate LFPI, we detected significant upregulation of the cytoplasmic TDP-43 in the ipsilateral cortices of injured animals at 1 and 3 d after TBI, with decreased nuclear TDP-43 content only on the third post-injury day. TDP-43 cellular mislocalization has also been previously described in several studies in which different models of TBI, varying injury severities, and experimental animal species were used [21,44,45,46,48,49,50]. In the preclinical investigations, cytosolic TDP-43 accumulation was observed in the cortices of rodents at various time points after single TBI induction [45,46,48]. Our results in the LFPI model are similar to those presented by Wright et al. [48]. Using only immunofluorescence staining, they detected TDP-43 cytoplasmic translocation in the rat cortical neurons following single moderate LFPI, though at time points different from the ones we used. In the study by Tan et al. [46], protein levels of cytoplasmic and nuclear full-length TDP-43, as well as the expressions of its 35-kDa and 25-kDa fragments, both phosphorylated and non-phosphorylated, in the cortices of mice subjected to LFPI, were measured. The authors reported increased nuclear expression but no change in the levels of cytoplasmic TDP-43 at 1 day and 1 week post-injury, which differs from the results we obtained. This discrepancy could be because Tan et al. used “relatively mild” injury severity, which is different from our study. Accordingly, as far as we know, our study would be the first one in which TDP-43 expression in mice following single LFPI of moderate severity was quantified in the cortical as well as in the hippocampal tissue extracts.

What we also detected, in the ipsilateral cortices of mice following LFPI, is that the TDP-43 cytoplasmic accumulation was present not only in neurons, as was reported by Wright et al. [48], but also in the reactive microglial cells. This discovery is interesting since it was found that the TDP-43-mediated activation of microglia can cause a proinflammatory cascade that could promote motoneuron injury [59] and consequently suggests a role for TDP-43 in the cortical inflammation, i.e., secondary injury cascade following single TBI of moderate severity. 

NF-κB is a major transcription factor and its activation is important for neurogenesis and synaptic plasticity in neurons [25,27]; however, in glial cells, NF-κB signaling pathway activation is linked to pro-inflammatory responses [60,61,62]. In the ipsilateral cortices of mice with LFPI, we detected slightly lower cytoplasmic p65 protein expression at post-injury day 3, which is mostly in accordance with previously published complementary studies [28,29,30,32]. One possible reason why the Western blotting analyses did not reveal statistically significant differences in this protein’s levels between the experimental groups could be due to different patterns of p65 expression related to the exact cortical area. Namely, in the core of the injured cortical tissue, we detected mostly cells with nuclear p65 staining; however, in the pericontusional area, this was not the case, as namely cells with pronounced cytoplasmic p65 staining were observed in this penumbral part of the cortex. The significance of the p65 Western blotting results could have consequently been affected since the tissue collected for the analyses contained both core and the pericontusional tissue. 

Our results regarding the TDP-43 and NF-κB cytoplasmic expressions in the animals with a single moderate LFPI also correlate to the findings by Thammissety et al. [47], in which TDP-43 and NF-κB interaction was investigated in a mouse stroke model. Specifically, they found that the increased cytoplasmic translocation of TDP-43 in the ischemic cortices coincided with the higher expression of the phosphorylated p65 subunit of NF-κB after 3 d of reperfusion. This is the same time point at which we found cytosolic TDP-43 over-expression and the loss of nuclear TDP-43, concomitantly with a slight decrease in the cytosolic p65 content in the mice subjected to LFPI. Furthermore, double immunofluorescence staining analyses also suggest injury area and cell type-dependent differences in the expressions of the p65 subunit of NF-κB and TDP-43 in the traumatized mouse cortex. Notably, in the pericontusional brain region, increased cytoplasmic p65 levels coincided with the decrease in the nuclear TDP-43 expression in the same, glia-like cells, suggesting the cell-type dependent nature of the relationship between the TDP-43 and p65 protein, which has already been previously proposed [63]. 

Synaptic dysfunction is one of the pathophysiological hallmarks of TBI and numerous chronic neurodegenerative disorders, such as AD, in which synapse loss is not always concomitant with neuronal death, but can also happen in surviving neurons [64]. PSD-95, along with other synaptic proteins, such as synaptophysin or growth-associated protein-43, has a significant role in maintaining synaptic plasticity, and its loss in the hippocampus has been associated with cognitive deficits observed following TBI as well as in mild cognitive impairment and AD [65]. In the mouse ipsilateral cortices, we detected a decrease in the PSD-95 expression at both 1 and 3 d after LFPI, which is probably due to the significant cortical neuronal loss characteristic of this type of brain injury. This result is in accordance with the previously demonstrated loss of synaptic PSD-95 in both immature and mature rodents following TBI [66,67,68,69,70,71]. We also found that, in the moderately brain-injured mice, in the cortical cells, pronounced cellular expression of PSD-95 positively correlated with an increased presence of cytoplasmic TDP-43, which suggests that these two proteins are crucial for the post-injury neuroplastic changes and thus are part of the physiological neuronal tissue response to a serious brain injury [40]. To our knowledge, our study is the first one to examine the relationship between the expressions of TDP-43 and PSD-95 in the experimental TBI in vivo. 

The victims of TBI often suffer from lasting cognitive deficits, memory difficulties, and behavioral disturbances, and increasing evidence suggests that, even without the presence of evident morphological changes, subtle physiological changes that occur in the hippocampus after TBI are of potentially great importance [72]. Among those pathophysiological changes, post-traumatic neuroinflammation plays an important part. Results of our analyses of the hippocampal changes following LFPI and rmTBI suggest differences in the importance of the TDP-43 pathology, which could be related to the post-traumatic sequelae. Namely, in the LFPI animals, we discovered that at 14 d following trauma there is a marked increase in the hippocampal presence of pathological forms of TDP-43. No such finding was revealed in the mice from the rmTBI experiment. This result is in contrast to the data from some clinical studies of single and repetitive TBI which also suggest a difference in the TDP-43 pathology [43,51]. Namely, the occurrence of cytoplasmic aggregates containing abnormally phosphorylated TDP-43 (a pathological hallmark of FTLD, ALS, and a common finding in other neurodegenerative diseases, such as the AD) was observed postmortem only in the brains of patients which endured repeated concussions during life [51]. Contrary to that, increased cytoplasmic levels of phosphorylation-independent TDP-43 were detected in both acute and long-term survivors of a single TBI [43], suggesting a physiological role for TDP-43 in response to this type of injury. We propose that these discrepancies between the clinical study by Johnson et al. and our research are related to the diverse mechanics of TBIs suffered by patients and different time points at which the analysis was done in the mentioned study. The LFPI model we used results in a more “mixed” type of injury, containing elements of both focal and diffuse tissue damage, including the elements of diffuse axonal injury. Accordingly, we suggest that the presence of pathologically phosphorylated TDP-43 in the hippocampus might be correlated to the occurrence and the extent of post-TBI axonal injury. Additionally, growing evidence suggests that TDP-43, similarly to prion proteins and transsynaptic spread of the hyperphosphorylated tau, possibly propagates and acts through axonal pathways [73]. Consequently, it could be argued that the detected subacute hippocampal TDP-43 pathology is the result of the transaxonal spreading. 

In our experiments, at 14 d following LFPI induction, the presence of the pathological TDP-43 species in the hippocampi of injured animals was not accompanied by changes in the microglial activation. Conversely, in mice subjected to repeated concussions and sacrificed at 14 d after the last mild trauma, we discovered some morphological changes pointing to microglial activation, but with no signs of TDP-43 proteinopathy. Taken together, these results indicate that, in this brain region and at the investigated time point, the TDP-43 protein expression pattern is not related to neuroinflammation. 

In this study, we did not consider the effects of the biological variables, such as the animal sex, or the investigation bias, e.g., regarding the judgment in cell counting. Moreover, we did not observe behavioral effects and differences in these subacute time points.

In conclusion, the present study demonstrated that single moderate TBI causes acute changes in the subcellular TDP-43 expression levels in the ipsilateral cortices of injured animals and a decrease in the cortical PSD-95 levels. PSD-95 expression correlated with the presence of increased cytoplasmic TDP-43 expression in the surviving cortical neurons acutely following LFPI. At 14 d post-LFPI, we also detected some pathological changes of the hippocampal TDP-43, which was not detected in the same brain region in mice following repeated mild brain traumas. Variances in the microglia activity in the hippocampus between the two models of TBI also indicate that there are some differences in the pathophysiological processes employed after different types of brain injury, which might in turn prove to be important for the prognosis and the treatment of TBI.

In our future investigations, we plan to examine the molecular background of observed TDP-43 mediated changes, specifically in relation to the expression of TBI specific inflammatory response, changes in different miRNAs expression profiles and the activation of MAPK signaling pathways.

## 4. Materials and Methods

### 4.1. Animals 

Experiments were performed on adult male wild type C57BL/6 mice (total number of animals *n* = 50). Male animals were chosen for this study in order to exclude the possible effects of different levels of female sex hormones released during the estrus cycle [74,75]. Moreover, male animals were chosen because studies have showed that, in the young adult population, males are more susceptible (55–80%) to sustain TBI than females. One of the reasons for this could be that, at this age group, males are significantly more involved in traffic injuries, violent activities, and sports injuries than females [76]. Animals were procured from the Laboratory of Mouse Engineering and Breeding Facility (LAMRI), University of Rijeka, Faculty of Medicine, at least 5 d prior experimental traumatic brain injuries [77]. Maintenance of the animals and the experiments was done in the animal facility located at the Department of Basic and Clinical Pharmacology and Toxicology, Faculty of Medicine, University of Rijeka. Mice were maintained under a 12 h light/dark cycle with water and food available ad libitum, at 22 ± 2 °C and 55 ± 10 % relative humidity, and were randomly divided in control or trauma groups by blind laboratory staff. Experiments were performed between 0800 and 1700. Animals were group housed 3–5 per cage for LFPI and rmTBI group. Normothermic body temperature of the mice subjected to the LFPI brain trauma was maintained with a heating lamp directly above the mouse and the body was covered with a blanket during surgical procedures. Maintenance of normothermia in the rmTBI group was not necessary because trauma procedure is rapid. After LFPI procedure, mice were kept in individual cages to facilitate their recovery. Experiments were designed to minimize the number of animals used, and in compliance with current institutional, national (NN 135/06, 37/13, 125/13, 55/13, 39/17) and international (European Parliament Directive 2010/63/EU) guidelines regulating the use of animals in the experimental studies. The experiments were approved by the Faculty’s Ethical and Animal Welfare Committees as well as the national regulatory body responsible for issuing ethical approval (Croatian Ministry of Agriculture).

### 4.2. Experimental Traumatic Brain Injuries

Single TBI of moderate severity was induced using the lateral fluid percussion injury (LFPI) apparatus, adjusted to the mouse [78,79]. In brief, mice were anesthetized with isoflurane (3.5% induction, 1.2–1.5% maintenance), their heads were mounted on the stereotactic apparatus (David Kopf Instruments, Tujunga, CA, USA), and the lubricant eye drops were applied. Craniotomy was performed above the left parietal cortex, 2.3 mm laterally to the midline, midway between lambda and bregma sutures. A modified Luer Lock needle hub, with an inside diameter of 2.5 mm, was fixed over the intact dura with dental acrylic glue and cement. LFPI device (VCU Biomedical Engineering Facility, Richmond, VA, USA) was connected to the Luer Lock with a 50 cm tube (ref. no. 8255172; B. Braun, Melsungen, Germany). The impact characteristics were measured on a pressure transducer and recorded on the oscilloscope. Single TBI of moderate severity was induced with a pressure pulse of 50 ms duration. Immediately after brain injury, mice were subjected to short anesthesia for stitching. Sham animals, used as the control group, underwent the same procedures but did not receive any head injury. The exclusion criteria after single LFPI/Sham injury was dura mater damage; however, in our experiment, all the animals had uncompromised dura mater. After single moderate trauma or sham injury, mice were returned to their cages for recovery. Respiratory problems or seizures were not observed.

**Figure 6:**
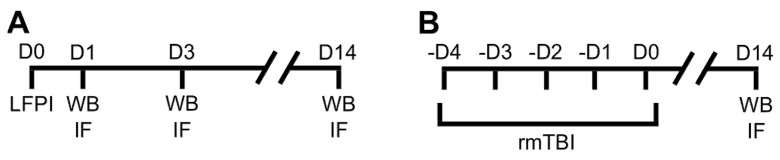
Experimental design and timeline of the experiments on mice subjected to (**A**) lateral fluid percussion injury (LFPI) of moderate severity or (**B**) repetitive mild traumatic brain injury (rmTBI). rmTBI group of mice was subjected to mild weight drop injuries twice daily, 5 d in a row. Mice of the LFPI group were sacrificed 1, 3, or 14 d after the injury, and animals exposed to rmTBI were euthanized at 14 d after the last head impact. Sham injured mice of the control groups for both LFPI and rmTBI experiments were sacrificed on day 1 after the experimental procedures. Different cohorts of mice were used for the Western blot (WB) and the immunofluorescent analyses (IF).

Repetitive mild brain trauma was induced in mice using the weight drop model as described by Kane et al. [80] and used in our previous research [21]. Briefly, for each mild injury, animals were lightly anesthetized with isoflurane (3.5%) and positioned on the nicked aluminum foil placed on the upper side of a Plexiglas box. Apparatus used for the head injury induction consists of a vertical metal tube (1 m) and a steel weight (97 g, 1.2 cm in diameter) that was suspended on a nylon thread. Weight was set just above the mouse’s head, then pulled up to 1 m height and released. In this type of injury, because of the mechanical force caused by the dropping weight, the animal breaks through the aluminum foil, rotates by 180°, and falls on the foam placed on the bottom of the Plexiglas box. In our experimental protocol, mild TBIs were induced twice a day, with daily 6 h intervals, 5 d in a row. As in the LFPI group, sham-treated animals were used as the control group, i.e., underwent the same procedures, but did not receive head traumas. After each mild trauma or sham injury, mice were returned to their cages for recovery. Respiratory problems or seizures were not observed. The timelines of all the animal experimental procedures are presented in detail in Figure 6.

### 4.3. Tissue Collection

Animals from the LFPI experiments were sacrificed at 1, 3, or 14 d after the single moderate TBI while the mice from the rmTBI protocol were euthanized at 14 d after the last mild brain trauma. All the sham injured mice, used as the respective control groups for the LFPI and the rmTBI experiments, were sacrificed 1 day after the end of the experimental procedures. We decided to use this single time point after sham TBI procedures by considering the results of our previous research using the LFPI model in rodents as well as the results of our preliminary studies of rmTBI in mice. Namely, in our previous experiments, we found no significant differences between the control animals sacrificed at different time points following sham procedures, which is something that can be also supported by a number of previously published studies [46,81,82]. For the Western blotting analyses in the LFPI experiment, ipsilateral and contralateral parietal cortices were dissected because, in this TBI model, that is the brain region in which the most serious tissue injury is found [52]. For the examination of changes in a region distant from the main injury site, the ipsilateral hippocampus for the LFPI and both hippocampi for the rmTBI experiments were dissected. Sampled cortices and the hippocampi were quickly obtained from the brains of the decapitated animals, frozen in liquid nitrogen, and stored at −80 °C until the analyses.

Brains from the separate cohorts of mice were used for immunohistological assays. For that purpose, at the same time points (Figure 6), mice were anesthetized with a mixture of ketamine and xylazine (100 mg/kg and 10 mg/kg, respectively), i.p. and transcardially perfused with ice-cold, phosphate-buffered saline (PBS, 0.1 M, pH 7.4) followed by 4% paraformaldehyde (PFA) in PBS. Brains were postfixed in 4% PFA overnight, dehydrated in 20% sucrose in PBS for 24–72 h and then snap-frozen in the tissue freezing medium (2-methyl butane) cooled in liquid nitrogen. Coronal cerebral cryosections were cut to a 10 μm thickness, dried for 3 h at 50 °C and, after achieving room temperature (RT), stored at −80 °C. 

### 4.4. Western Blotting

Cytosolic and nuclear fractions from the isolated cortices and the hippocampi were prepared for the detection of the designated proteins’ expression levels by Western blotting technique, as previously described by Yadavilli et al. [83]. Briefly, samples were first washed with PBS and centrifuged at 3000 rpm for 5 min at 4 °C and afterward were subjected to a series of centrifugations with lysis buffers containing enzyme inhibitors. First pellet was resuspended with lysis buffer A (25 mM HEPES [pH 7.4], 50 mM KCl, 2 mM EDTA, 2 mM EGTA, 0.1% Triton-X, 1 mM DTT, 25 mM NaF, 1 mM NaVO_3_, 1 mM PMSF, 10 µg/mL aprotinin, 10 µg/mL leupeptin and 10 µg/mL NaPP), homogenized in Dounce homogenizer and incubated for 30 min on ice. After centrifugation at 13,375 rpm for 5 min at 4 °C, the supernatant was collected as the cytosolic fraction and stored at −80 °C. Pellet was resuspended with lysis buffer B (buffer A without Triton-X) and centrifuged at 3000 rpm for 5 min at 4 °C. The supernatant was thrown away and the pellet was resuspended in the nuclear buffer (buffer B with 450 mM KCl and 50% glycerol) and frozen for 30 min at −80 °C. After thawing at RT, the sample was centrifuged at 13,375 rpm for 15 min at 4 °C, and the supernatant was collected as the nuclear fraction and, finally, stored at −80 °C. 

Equal amounts of proteins per lane were loaded on a sodium dodecyl sulfate-polyacrylamide gel, separated by electrophoresis, and then transferred to nitrocellulose membranes. According to the antibodies’ manufacturers’ recommendations, membranes were blocked in 5% non-fat dry milk or bovine serum albumin (BSA) as the blocking buffers for one hour at RT. The incubation of the membranes with different primary antibodies, as shown in Table S1, was performed overnight at 4 °C. On the next day, membranes were incubated in the appropriate biotinylated secondary antibody solutions (Table S1) for 1 h at RT, followed by streptavidin–horseradish peroxidase conjugate incubation for half an hour. Blots were developed using the Super Signal West Pico Chemiluminescent Substrate (Thermo Fisher Scientific, Waltham, MA, USA). Signal was detected by the Kodak Image Station 440CF and the quantification of the band intensities was performed with the Kodak 1D Image Analysis Software (Eastman Kodak, Rochester, NY, USA). For the figures that include representative Western blots, original blot images are provided in the supplementary file.

### 4.5. Immunofluorescence, Histochemistry and Imaging

Frozen brain sections were left to achieve RT and then dried at 50 °C for 10 min. Permeabilization was done with tris-buffered saline (TBS) with 0.025% Triton X-100, for 10 min at RT. Blocking buffer made of 5% normal serum (goat, chicken, or rabbit, depending on the secondary antibody used afterward) was prepared in 1% BSA/TBS-Triton X-100 (0.025%), and applied for 2 h at RT. Primary antibodies, according to Table S1, were administered overnight at 4 °C. On the next day, a fluorochrome-conjugated secondary antibody (shown in Table S1) or biotinylated secondary antibody was applied for 1 h. If the section was treated with biotinylated secondary antibody, streptavidin-conjugated with the appropriate fluorochrome (Thermo Fisher Scientific, Waltham, MA, USA) was applied for 20 min. 4′,6-diamidino-2-phenylindole (DAPI) (Invitrogen, Carlsbad, CA, USA) was used to stain the nuclei. After washing in the distilled water, sections were embedded in Mowiol (Sigma–Aldrich, St. Louis, MO, USA) and coverslipped. Microphotographs of the sections were taken using the Olympus BX 51 microscope, equipped with the Olympus DP 70 digital camera (Olympus, Tokyo, Japan) and taken with cellSens software (Olympus, Tokyo, Japan).

Cresyl violet staining (Nissl staining) was used for identifying the basic neuronal structure and the brain injury extent. Frozen sections were left to achieve RT and afterwards were dried at 50 °C for 10 min. Slides were immersed directly into 1:1 alcohol/chloroform overnight and were then rehydrated through 100% and 95% alcohol to distilled water. Staining of the slides was performed with warmed acidic 0.1% cresyl violet solution for 10 min. Slides were then rinsed in distilled water and, after differentiation in 95% ethyl alcohol, dehydrated in 100% alcohol and cleared in xylene. Mounting was performed with Entellan (Merck Millipore, Bedford, MA, USA).

In the animals from the LFPI and the rmTBI experiment, microphotographs of hippocampal sections immunolabeled with a primary antibody raised against ionized calcium-binding adaptor molecule (Iba1) were used for the quantitative analysis of microglia morphology, as previously described by Morrison et al. [84], in which ImageJ software (National Institute of Health, Bethesda, MD, USA) and appropriate plugins were used. Briefly, for the skeleton analysis, at least six epifluorescence microphotographs per animal of the hippocampal dentate gyri (from the brain sections cut at bregma from −0.45 to −1.85) were imaged at x400 magnification and the regions of interest (an area approximately 0.037 mm^2^) were selected. Images were converted to the binary format, processed using the appropriate ImageJ plugins (i.e., contrast, unsharp mask, despeckle, threshold, close, remove outliers), and then used for the determination of the process length and the number of endpoints/processes per Iba1 positive cell by blind investigator. In the same microphotographs, the number of Iba1 positive cells in the dentate gyrus of the hippocampus was counted. 

### 4.6. Statistical Analyses

Data were collected using Microsoft Excel 2016 (Microsoft Corp., Redmond, WA, USA). For the densitometric analyses of the expression levels of different proteins, the values of the relative optical densities of the bands were corrected for the corresponding β-actin (cytosolic samples) or histone-H3 (nuclear samples) contents and were then expressed as the % of the values from the related control groups. For all the statistical analyses, Statistica software version 13.0 (StatSoft, Inc., Tulsa, OK, USA) was used. Sample size, i.e., the number of animals per group for the Western blotting and the immunofluorescence analyses, was calculated by using the results of our previous research in which complimentary methods were used [21,85]. Namely, we have conducted similar studies in the past, investigating the effects of rmTBI on the expression levels of different proteins in the mouse cortex. Using the means from this study for different investigated parameters, we calculated the minimum number of animals per group [21]. A comparison between two independent groups was achieved by Student’s unpaired *t*-test and comparisons between multiple groups were done using one-way analysis of variance (ANOVA) followed by Duncan’s post-hoc multiple comparison test. Results are expressed as mean ± standard deviation (SD). In all the comparisons, *p* < 0.05 was considered to indicate statistical significance. To remove the variations between the Western blotting sessions, results of the densitometric analyses were corrected with the Factor Correction program version 16.1 [86].

## Data Availability

The data that support the findings of this study are available within the article and supplemental data or upon request from the corresponding author.

## Supplementary Materials

The following are available online at https://www.mdpi.com/article/10.3390/ijms222212211/s1.
