# Supplementary material for: Differential Expression Patterns of TDP-43 in Single Moderate versus Repetitive Mild Traumatic Brain Injury in Mice
Source: Int J Mol Sci. 2021 Nov 11;22(22):12211. doi: 10.3390/ijms222212211 (PMC8621440; doi:10.3390/ijms222212211)

International Journal of *Molecular Sciences*

MDPI

Table S1. List of antibodies used for the western blotting (WB) and the immunofluorescent (IF) analyses.

Primary antibody	Structure of immunogen	Manufacturer; catalog number; host species	Concetration
anti-TDP-43	TDP-43 fusion protein	Proteintech; 10782-2-AP; rabbit polyclonal	1:1000 (WB)<br>1:200 (IF)
anti-NF-κB p65	amino acids 1-286 mapping at the N-terminus of NFκB p65 of human origin	Santa Cruz Biotechnology; sc-8008; mouse monoclonal	1:500 (WB)<br>1:50 (IF)
anti-PSD-95	amino acids 50-150 (internal sequence) conjugated to keyhole limpet haemocyanin (Cysteine residue)	Abcam; ab18258; rabbit polyclonal	1:1000 (WB)<br>1:100 (IF)
anti-β-actin	β-actin (C4) raised against gizzard Actin of chicken origin.	Santa Cruz Biotechnology; sc-47778; mouse monoclonal	1:1000 (WB)
anti-histone H3	Synthetic peptide corresponding to Human Histone H3 aa 100 to the C-terminus conjugated to keyhole limpet haemocyanin	Abcam; ab1791; rabbit polyclonal	1:1000 (WB)
anti-NeuN	NeuN clone (A60) Purified cell nuclei from mouse brain	Merck Millipore; MAB377; mouse monoclonal	1:100 (IF)
anti-Iba1	Synthetic peptide corresponding to Human Iba1 aa 135-147 (C terminal)	Abcam; ab5076; goat polyclonal	1:500 (IF)<br>1:2000 (WB)
anti-GFAP	total GFAP protein (full lenght)	Cell Signaling Technology; 3670; mouse monoclonal	1:200 (IF)
anti-phospho (409/410)-TDP-43	Phospho-TDP43 (Ser409/410)	Proteintech; 22309-1-AP; rabbit polyclonal	1:1000 (WB)
anti-TLR2	Recombinant fragment. Immunogen information is proprietary to Abcam	Abcam; ab209217; rabbit monoclonal	1:1000 (WB)
anti-iNOS	Synthetic peptide corresponding to Mouse iNOS aa 1-100	Abcam; ab3523; rabbit polyclonal	1:1000 (WB)
anti-CD86	Tissue, cells or virus corresponding to Human CD86. ARH-77 (B-lymphoblastoid cell line).	Abcam; ab213044; mouse monoclonal	1:1000 (WB)
anti-CD206	Synthetic peptide conjugated to KLH derived from within residues 1400 to the C-terminus of Human Mannose Receptor.	Abcam; ab64693; rabbit polyclonal	1:1000 (WB)
**Secondary antibody**		**Manufacturer; catalog number; host species**	**Dilution**
biotinylated goat anti-mouse	Gamma Immunoglobins Heavy and Light chains	Thermo Fisher Scientific; A16070; goat polyclonal	1:3000 (WB)<br>1:200 (IF)
biotinylated goat anti-rabbit	Gamma Immunoglobin	Thermo Fisher Scientific; 65-6140; goat polyclonal	1:2000 (WB)<br>1:200 (IF)
goat anti-rabbit Alexa Fluor 488	Gamma Immunoglobins Heavy and Light chains	Thermo Fisher Scientific; A-11034; goat polyclonal	1:200 (IF)
chicken anti-goat Alexa Fluor 594	Gamma Immunoglobins Heavy and Light chains	Thermo Fisher Scientific; A-21468; chicken polyclonal	1:200 (IF)
chicken anti-rabbit Alexa Fluor 488	Gamma Immunoglobins Heavy and Light chains	Thermo Fisher Scientific; A-21441; chicken polyclonal	1:200 (IF)

Abbreviations: TDP-43, TAR DNA binding protein 43; NF-κB p65, nuclear factor kappa-light-chain-enhancer of activated B cells p65 subunit; PSD-95, postsynaptic density protein 95; NeuN, neuronal nuclei protein; Iba1, ionized calcium-binding adapter molecule 1; GFAP, glial fibrillary acidic protein; phospho(409/410)-TDP-43, phosphorylated TDP-43; TLR2, Toll-like receptor 2; iNOS, inducible nitric oxide synthase; CD86, cluster of differentiation 86; CD206, cluster of differentiation 206.

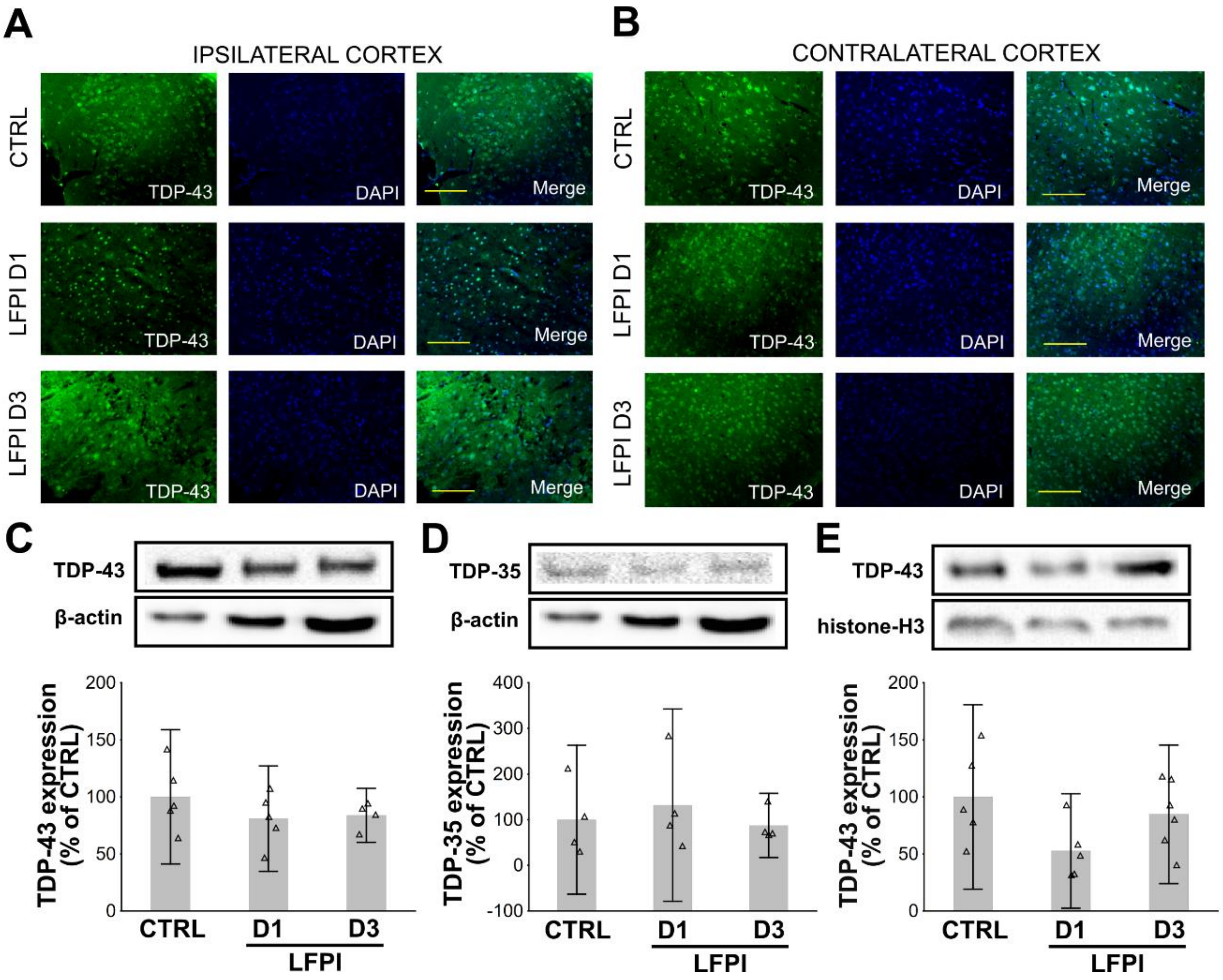

Figure S1. Localization of the transactive response (TAR) DNA binding protein 43 (TDP-43) in the mouse ipsilateral and contralateral cortices at 1 and 3 days after single moderate lateral fluid percussion injury (LFPI). **(A-B)** Representative microphotographs of the **(A)** ipsilateral and **(B)** contralateral cortices labelled with anti-TDP-43 antibody (green) and counterstained with DAPI nuclear stain (blue) in the animals of the control group (CTRL) and the mice sacrificed 1 (LFPI D1) or 3 (LFPI D3) days following single moderate LFPI. Scale bar: 100 μm. **(C-E)** Representative immunoblots of TDP-43, β-actin and histone-H3 and the bar plots of their corresponding densitometry analyses of the cytoplasmic **(C)** and the nuclear **(E)** TDP-43 expression levels of the contralateral cortex of the animals of the control group (CTRL) and the injured mice sacrificed at different time points following LFPI. Additionally, an analysis of the cytoplasmic TDP-35 expression was done **(D)**. In the densitometric analyses, TDP-43/TDP-35 results were corrected for the values of β-actin (cytoplasmic loading control) or histone-H3 (nuclear loading control) and expressed as % of the related control groups. Error bars represent ± SD (n=4-6 mice per group). Each triangle represents data from an individual mouse.

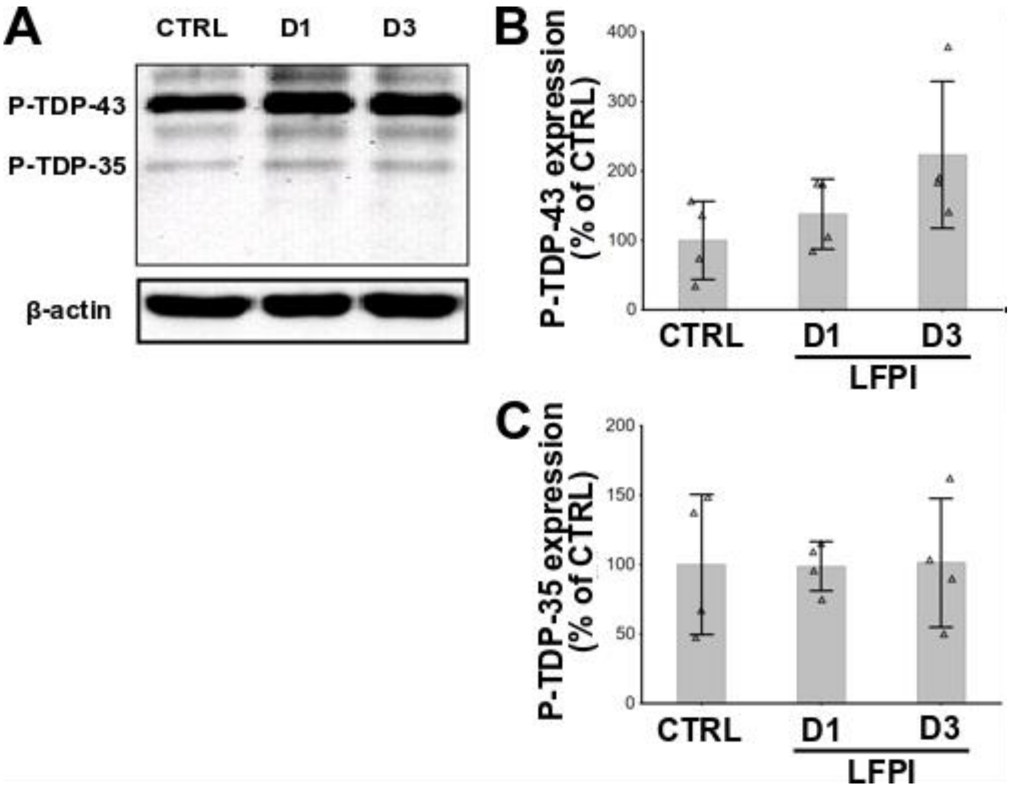

Figure S2. Effects of single moderate lateral fluid percussion injury (LFPI) on the levels of phosphorylated TDP-43 species in the mouse cortex at 1 and 3 days after the brain trauma. **(A)** Representative immunoblots of the phosphorylated TDP-43 (P-TDP-43), TDP-35 (P-TDP-35) and β-actin and bar plots of the corresponding densitometric analyses **(B, C)** of the cortical cytoplasmic expression levels. In all the densitometric analyses, the results were corrected for the values of β-actin (cytoplasmic loading control) and expressed as % of the related control group. Error bars represent ± *SD* (n=3-4 mice per group).

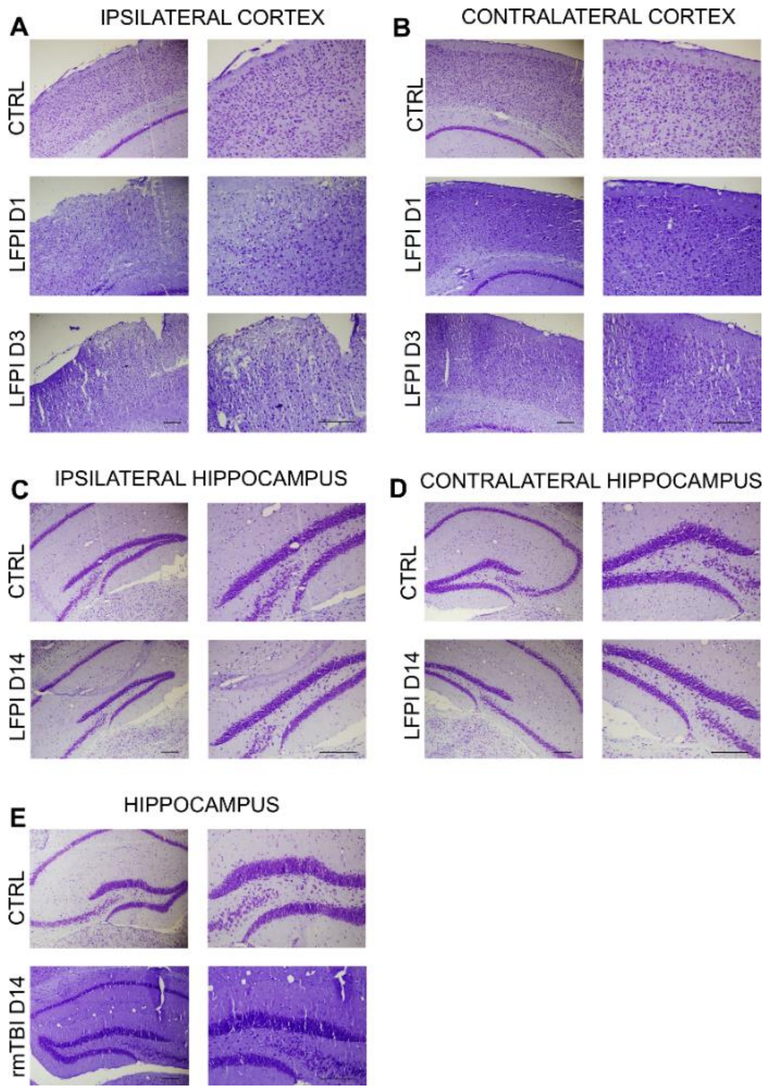

Figure S3. Histological changes after single moderate lateral fluid percussion injury (LFPI) or repetitive brain injury (rmTBI) in mice. **(A-E)** Representative microphotographs of the **(A)** ipsilateral and **(B)** contralateral cortices in the animals of the control group (CTRL) and the mice sacrificed 1 (LFPI D1) or 3 (LFPI D3) days following single moderate LFPI. Representative microphotographs of the **(C)** ipsilateral and **(D)** contralateral hippocampus of the animals of the control group (CTRL) and the mice sacrificed 14 (LFPI D14) days following single moderate LFPI. **(E)** Representative microphotographs of the hippocampus of the animal of the rmTBI control group (CTRL) and the mouse sacrificed 14 days after the last rmTBI (rmTBI D14). Scale bar: 200 μm. Evident pathological changes were observed in the ipsilateral cortex of the mouse subjected to LFPI in comparison to the animal of the control group, as the ipsilateral cortex is the brain region situated directly below the site of trauma application. Major histological differences between the ipsilateral and contralateral hippocampi in the mice of the control and LFPI groups were not visible. Also, major histological differences in the hippocampus of the rmTBI compared to its control were not observed.

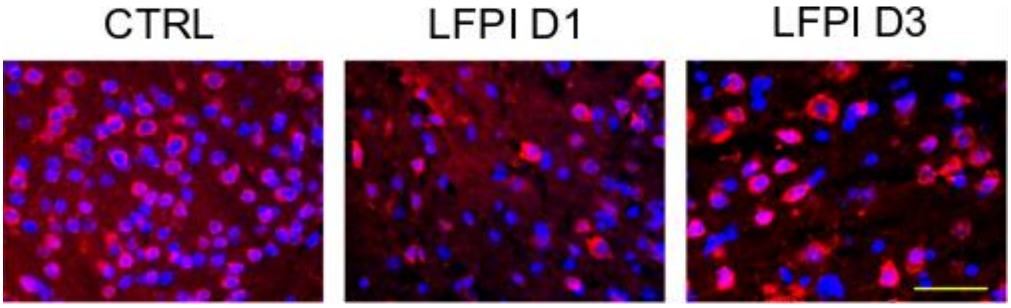

Figure S4. Neuronal marker (NeuN) stained sections of the mouse cortices after the single moderate lateral fluid percussion injury (LFPI). Representative microphotographs show NeuN immunostained (red) sections, counterstained with DAPI nuclear stain (blue) in the animals of the control group (CTRL), sacrificed 1 day after the sham injury, and the mice sacrificed 1 (LFPI D1) or 3 (LFPI D3) after the brain trauma. Figures are representative for N = 4 animals/group. Scale bar: 100 μm.

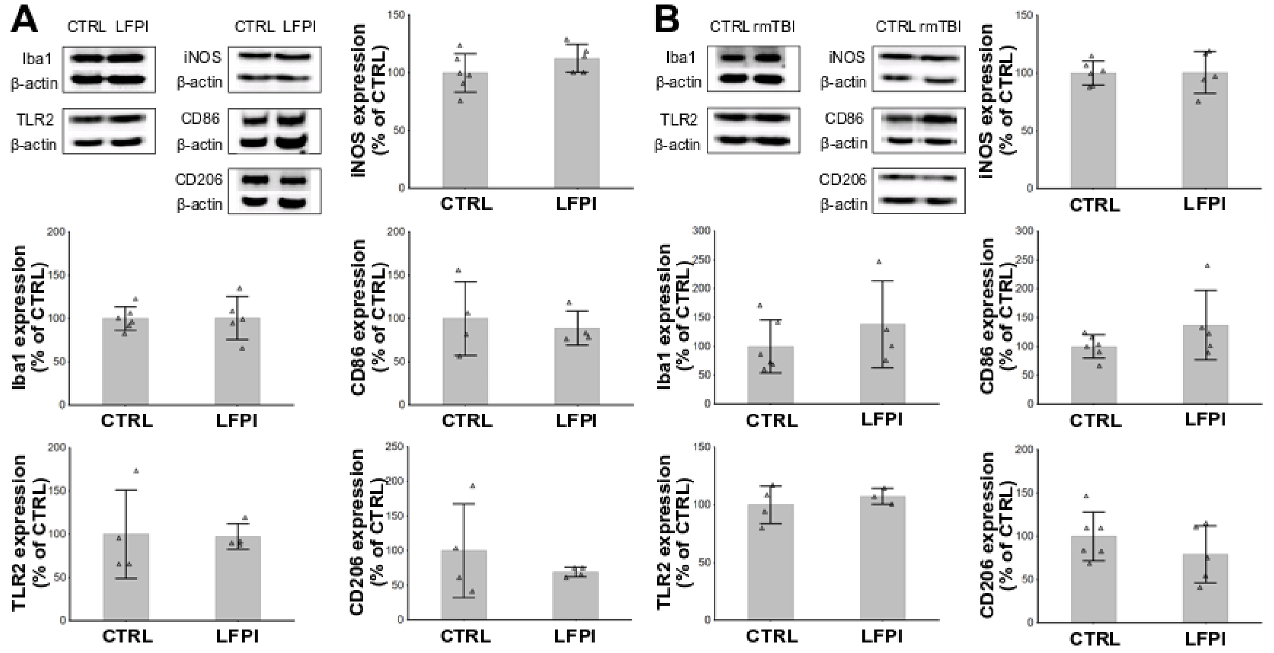

Figure S5. Single moderate lateral fluid percussion injury (LFPI) or repetitive mild traumatic brain injury (rmTBI) do not cause changes in the hippocampal microglia activation markers 14 days after the experimental traumatic brain injury protocols. **(A, B**) Representative immunoblots of microglial markers for ionized calcium binding adaptor molecule (Iba1), Toll-like receptor 2 (TLR2), inducible nitric oxide synthase (iNOS), cluster of differentiation 86 (CD86), cluster of differentiation 206 (CD206) and β-actin, and bar plots of their corresponding densitometric analyses of the cytoplasmic expression levels in the animals of the related control groups (CTRL) and mice sacrificed at 14 days after (**A**) LFPI or (**B**) the last mild repetitive trauma. Neither LFPI nor rmTBI caused a significant change in the levels of aforementioned microglial markers. In all the densitometric analyses, the results were corrected for the values of β-actin (cytoplasmic loading control) and expressed as % of the related control groups. Error bars represent ± *SD* (n=3-6 mice per group).

Original Western blots referring to the figures in the manuscript and the supplementary file

Red line boxes highlight the regions of the Western Blots shown in the figures.

All the Western blots were named according to the main figure to which they relate.

Figure S1 C, D

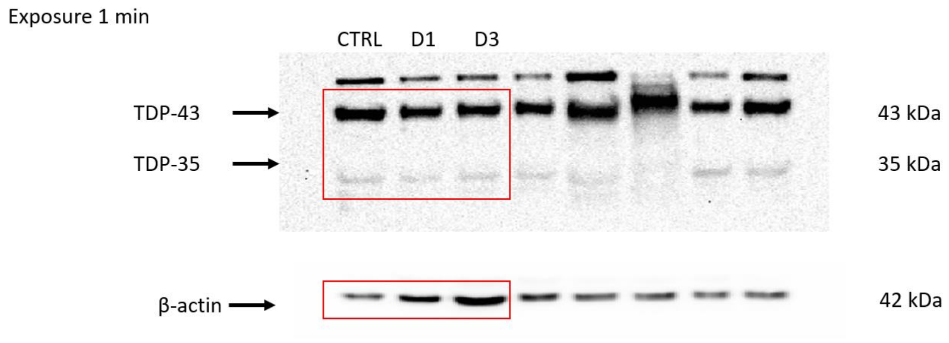

Figure S1 E

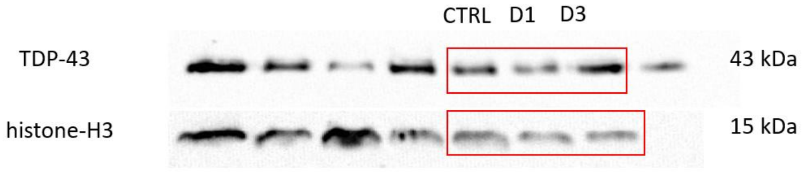

Figure S2

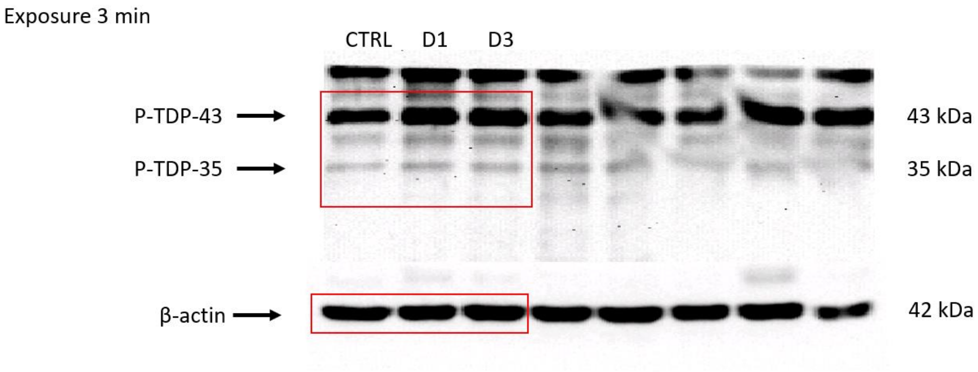

Figure S5A

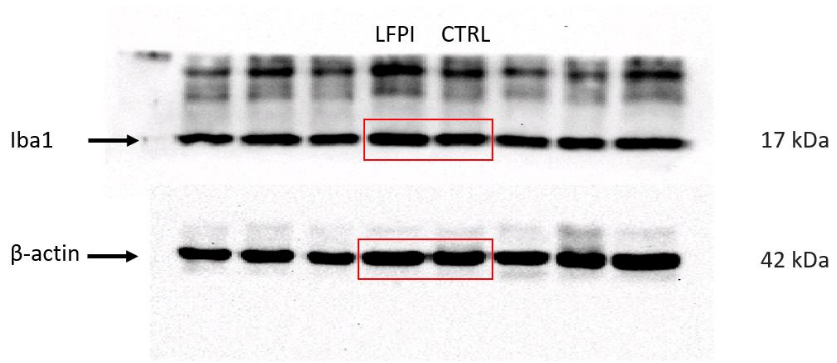

Figure S5A

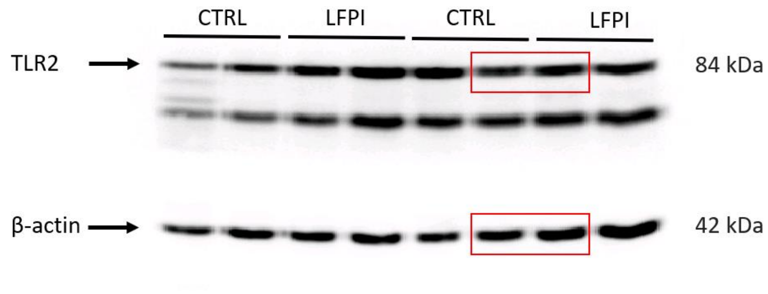

Figure S5A

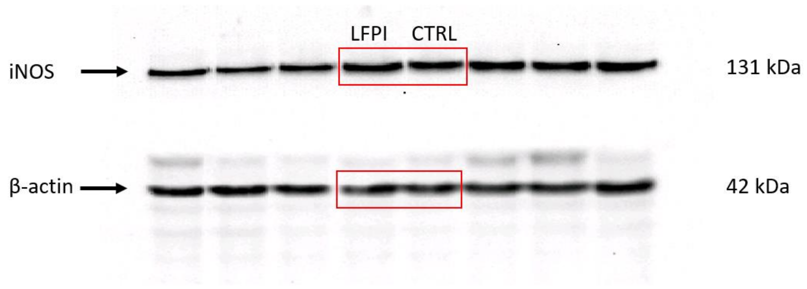

Figure S5A

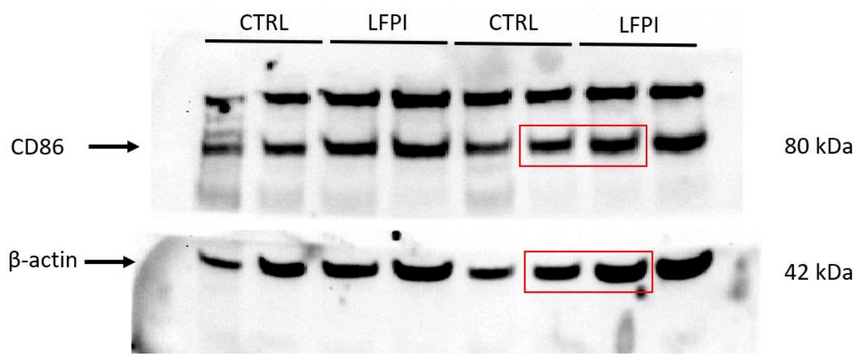

Figure S5A

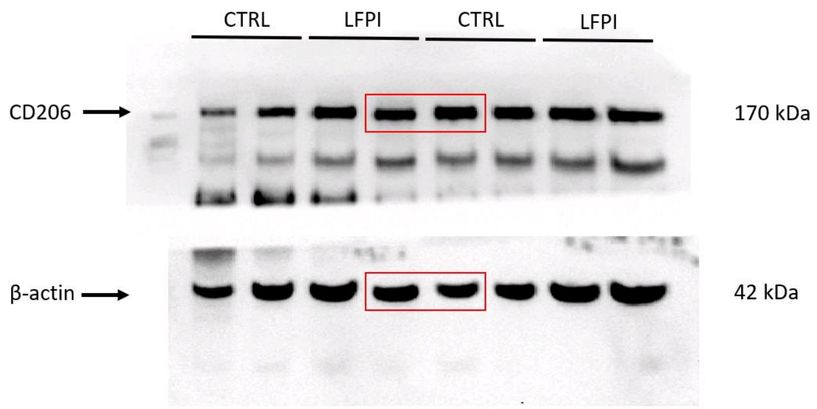

Figure S5B

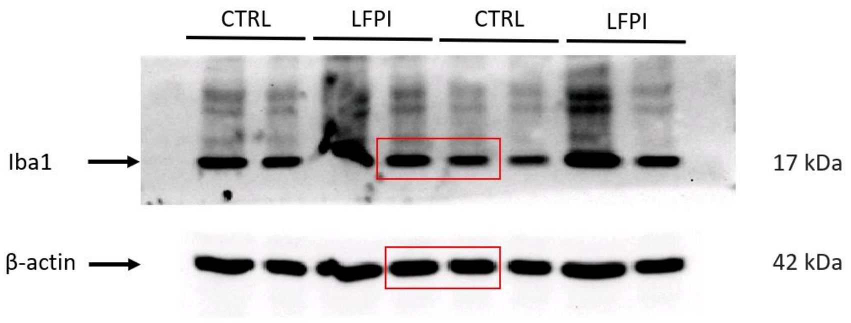

Figure S5B

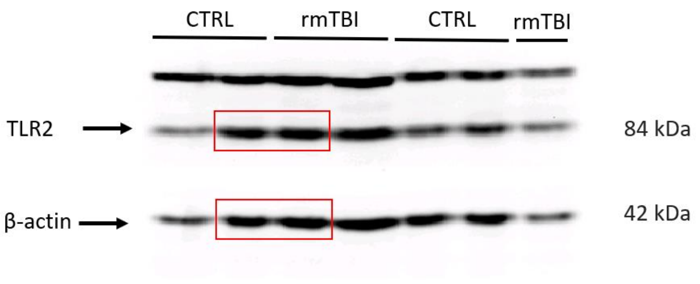

Figure S5B

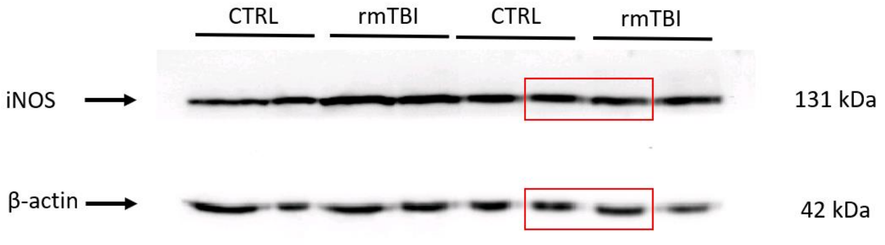

Figure S5B

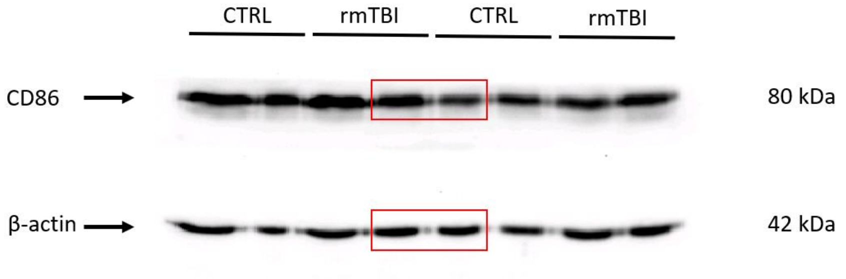

Figure S5B

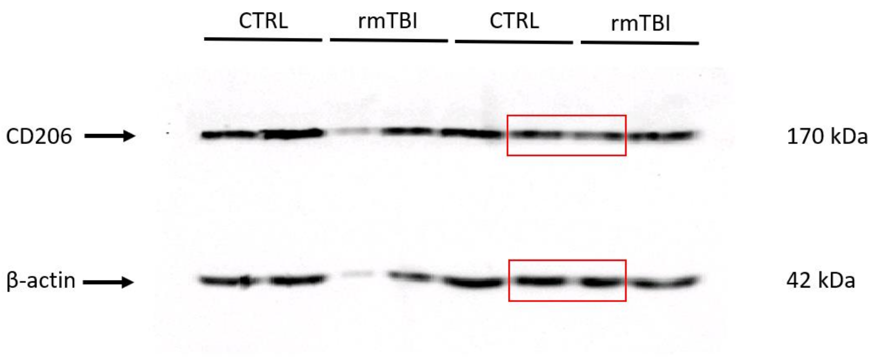

Supplement: Supplementary file 1 [file ijms-22-12211-s001.zip › ijms-1426553-SI-done.pdf]
